# Mosquito transcriptome changes and filarial worm resistance in *Armigeres subalbatus*

**DOI:** 10.1186/1471-2164-8-463

**Published:** 2007-12-18

**Authors:** Matthew T Aliota, Jeremy F Fuchs, George F Mayhew, Cheng-Chen Chen, Bruce M Christensen

**Affiliations:** 1 Department of Pathobiological Sciences, University of Wisconsin, 1656 Linden Drive, Madison, WI 53706 USA; 2 Department of Tropical Medicine, National Yang-Ming University, 155, Sec 2, Li-nun Street, Taipei 112, Taiwan

## Abstract

**Background:**

*Armigeres subalbatus *is a natural vector of the filarial worm *Brugia pahangi*, but it rapidly and proficiently kills *Brugia malayi *microfilariae by melanotic encapsulation. Because *B. malayi *and *B. pahangi *are morphologically and biologically similar, the *Armigeres-Brugia *system serves as a valuable model for studying the resistance mechanisms in mosquito vectors. We have initiated transcriptome profiling studies in *Ar. subalbatus *to identify molecular components involved in *B. malayi *refractoriness.

**Results:**

These initial studies assessed the transcriptional response of *Ar. subalbatus *to *B. malayi *at 1, 3, 6, 12, 24, 48, and 72 hrs after an infective blood feed. In this investigation, we initiated the first holistic study conducted on the anti-filarial worm immune response in order to effectively explore the functional roles of immune-response genes following a natural exposure to the parasite. Studies assessing the transcriptional response revealed the involvement of unknown and conserved unknowns, cytoskeletal and structural components, and stress and immune responsive factors. The data show that the anti-filarial worm immune response by *Ar. subalbatus *to be a highly complex, tissue-specific process involving varied effector responses working in concert with blood cell-mediated melanization.

**Conclusion:**

This initial study provides a foundation and direction for future studies, which will more fully dissect the nature of the anti-filarial worm immune response in this mosquito-parasite system. The study also argues for continued studies with RNA generated from both hemocytes and whole bodies to fully expound the nature of the anti-filarial worm immune response.

## Background

Mosquitoes are the most medically important arthropods for transmitting infectious diseases. The maintenance and spread of the pathogens that cause malaria, lymphatic filariasis, and a host of arboviral infections are all dependent on competent mosquito vectors. These diseases continue to have devastating effects, placing enormous health and economic burdens on less privileged populations of the tropical and subtropical regions of the world. This continued mortality and morbidity in these areas, along with the decreasing efficacy of traditional methods of vector-borne disease control, has forced the scientific community to develop new, more efficient means for vector control and treatment [1-3].

At the forefront of this exploration is the hypothesis that genetically engineered mosquitoes can be used to control mosquito-borne diseases [4-6]. Mosquitoes lack the adaptive immune mechanisms that provide vertebrates with pathogen-specific receptors and immune memory; therefore, innate immune responsiveness is of particular interest in such explorations because extensive research efforts have shown that mosquitoes can produce robust humoral and cellular immune responses against invading pathogens [7-11]. However, there is much to learn in understanding the innate immunity of mosquitoes in relation to invading pathogens. Until these processes are more completely understood, it could be extremely difficult to drive parasite resistance genes into wild, susceptible vectors in areas of endemic infection [12].

One such immune response drawing considerable attention is melanization. Melanization primarily plays a role during developmental stages such as cuticle formation, cuticle hardening between molts, and egg chorion tanning. It is a multi-enzymatic pathway that can also manifest as a response to cuticular wounding and to pathogens that invade the body cavity of the host. In addition, melanization functions as an essential component of the cell-mediated immune response in mosquitoes; however, very little is known about its genetic control [1,13,14].

In the mosquito *Armigeres subalbatus*, melanotic encapsulation functions as a natural mechanism of resistance to filarial worm invasion. Following ingestion of a blood meal, microfilariae (mf) penetrate the midgut and are rapidly melanized in the hemocoel. As soon as 10 minutes following a blood meal, melanin deposition is evident on the mf cuticle. At 12–16 hours post feeding, melanization is well underway and pathological effects on the mf are evident. At 24 to 48 hours post feeding mf begin to die, and by 72 hours post feeding, the response is all but complete [Christensen *et al*., unpublished].

When susceptible mosquitoes ingest *Brugia spp*. mf in a blood meal, parasites penetrate the midgut epithelium, enter the hemocoel, and migrate to the thoracic musculature, where development takes place. Once they have molted to the third juvenile stage they migrate through the hemocoel, eventually reaching the proboscis from which they escape when the mosquito feeds [15]. *Ar. subalbatus *is a natural vector of the filarial worm *Brugia pahangi*, but it kills *Brugia malayi *mf by melanotic encapsulation [16]. Because *B. malayi *and *B. pahangi *are morphologically and biologically similar, this mosquito-parasite system serves as a valuable model for studying resistance mechanisms in mosquito vectors [17].

Accordingly, we have initiated transcriptome profiling studies of *Ar. subalbatus *in relation to anti-filarial worm immune responses to clarify molecular components involved in *B. malayi *refractoriness. This represents the first holistic study conducted on the anti-filarial worm immune response that mimics a natural exposure to the parasite: both the number of parasites used to challenge mosquitoes and the way parasites were introduced into the mosquitoes (via blood feeding) in this study mimic the natural phenomenon [18], whereas previous studies usually have relied on unnatural amounts of mf or bacteria introduced into the hemocoel via intrathoracic injection to activate the melanization response [7,19,20]. The time course chosen facilitates a complete examination of the mosquito's transcriptome, beginning with the very start of the anti-filarial worm response and spanning to well after melanization has finished [16,21].

These initial studies assess the transcriptional response of *Ar. subalbatus *to infection with *B. malayi*. Herein, we demonstrate that the molecular components of the anti-filarial worm immune response include a number of unknown and conserved unknowns, cytoskeletal and structural components, and stress and immune responsive factors, indicating that the anti-filarial worm immune response in *Ar. subalbatus *is a highly complex process that involves the distinct effector response of melanization in concert with many other factors of both known and unknown function in order to clear filarial worms from the hemolymph. In addition, data show that these immune responses are extremely tissue specific, and when conducting studies of this nature, i.e. investigating a particular physiology, it is possible to under represent the desired transcriptional activity due to the disproportion created by using too general of an RNA source.

## Results

### Temporal Expression

**Figure 1:**
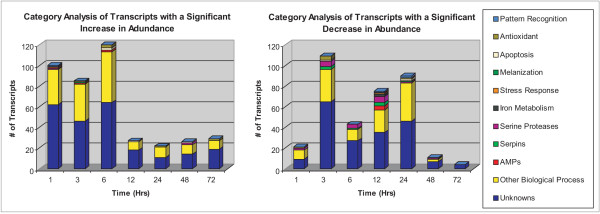
Functional composition of transcripts significantly affected by parasite challenge based on abundant and immunity-related EST clusters observed from *Ar. subalbatus *cDNA libraries. Transcripts with a detectable increase in abundance (top) and with a detectable decrease in abundance (bottom) 1, 3, 6, 12, 24, 48, and 72 hours after exposure to a *B. malayi *infective blood meal.

**Figure 2:**
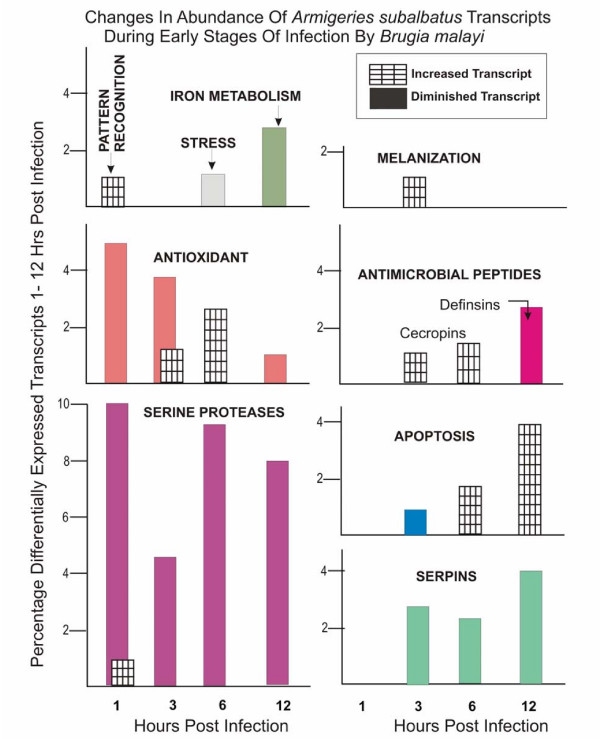
Further functional dissection of immunity-related transcripts significantly affected by parasite challenge at 1, 3, 6, and 12 hours post infection. Graphical representation of the percentage of immunity-related transcripts with a detectable increase or decrease in abundance (y-axis) at a given time (x-axis).

Volcano plots were used to create working gene lists to identify differentially expressed transcripts at each time point. At 1, 3, 6, 12, 24, 48, and 72 hours post challenge there were 121, 194, 163, 102, 111, 37, and 33 detectable transcripts, respectively, with significantly different transcriptional behavior (increased or decreased transcript abundance at a 95% confidence interval over two-fold values) as a result of parasite challenge (Figs 1 and 2). Between each time point there was very little overlap in the transcripts that showed significantly different transcriptional behavior (Table 1). For example, of the transcripts that significantly increased or decreased in abundance at 1 and 3 hours, only six were shared between the two time points, and this served as a common trend observed between time points, i.e. there were very few common features. The six shared transcripts were an unknown [GenBank:EU205545], a structural molecule [GenBank:EU209249], a serine protease [GenBank:EU205658], cathepsin [GenBank:EU208958], an ATP-dependent helicase [GenBank:EU212400], and a phosphoric monoester hydrolase [GenBank:EU209772]. This incongruity between the time points suggested that there is a great deal of informative and continual change in the transcriptome of *Ar. subalbatus *in response to infection with *B. malayi*. In addition, it is possible that additional transcripts were missed because the time points used may not have captured significant differences in transcript abundance for certain genes. For a full representation of all transcripts showing a detectable increase or decrease in abundance at all time points please see Additional File 1.

**Table 1:** Common features between time points.

	**1**	**3**	**6**	**12**	**24**	**48**	**72**
**1**	**121**	6	12	4	2	2	0
**3**	ø	**194**	25	26	5	1	0
**6**	ø	ø	**163**	24	2	3	6
**12**	ø	ø	ø	**102**	1	5	0
**24**	ø	ø	ø	ø	**111**	1	1
**48**	ø	ø	ø	ø	ø	**37**	0
**72**	ø	ø	ø	ø	ø	ø	**33**

Between each time point there was very little overlap in the transcripts that showed significantly different transcriptional behavior. This incongruity between the time points suggests that there is a great deal of informative and continual change in the transcriptome of *Armigeres subalbatus *in response to infection with *Brugia malayi*.

### Pattern Recognition

At 1 hour post infection, there was a detectable increase in the abundance of a peptidoglycan recognition protein transcript [GenBank:EU211663] that has probable immune and defense response activity (GO annotation). At 12 hours post infection, there was a detectable decrease in the abundance of a C-type lectin transcript [GenBank:EU207761] that had no previously described role in the anti-filarial worm defense response. Finally, at 24 hours post infection, there was a detectable decrease in the abundance of a transcript showing sequence similarity to an *Aedes aegypti *C-type lectin [GenBank:EU206532] with a probable role in immunity [22].

Also showing significantly different transcriptional behavior during filarial worm infection was calreticulin [GenBank:EU206649]. The abundance of calreticulin transcript in *Ar. subalbatus *responding to *B. malayi *infection showed no detectable change at 1, 3, 6, and 12 hours, but the transcript showed a detectable decrease in relative abundance at 24 and 48 hours post infection. By 72 hours it had returned to base line.

### Melanization

Of the transcripts implicated in the biosynthesis of melanin and melanization immunity included on our microarray (phenylalanine hydroxylase (PAH), dopachrome conversion enzyme (DCE), prophenoloxidase (ProPO1–4), etc), the only transcript to show differential transcription over controls was PAH [GenBank:EU206838]. The abundance of PAH transcript peaked at 3 hours and steadily declined through 6 and 12 hours. At 24 hours following an infective blood meal, a decrease in abundance of the transcript was evident. At 48 and 72 hours, the transcript showed no detectable change in abundance.

### Serine Proteases

We are most interested in those serine proteases that play a role in melanin biosynthesis and anti-microbial peptide (AMP) production because they are most likely involved in the anti-filarial worm immune response. There were 30 serine proteases or serine protease inhibitors (serpins) with significantly different transcriptional activity over the time course: 3 showed a detectable increase in transcript abundance and 27 showed a detectable decrease at specific time points. Increases in transcript abundance were detected for one serine protease transcript [GenBank:EU212480] at 1 hour and two [GenBank:EU206855 and GenBank:EU205695] at 72 hours following parasite challenge. Decreases in transcript abundance were detected for two serine protease transcripts [GenBank:EU205924 and GenBank:EU205658] at 1 hour, five [GenBank:EU205580, GenBank:EU206038, GenBank:EU205154, GenBank:EU212101, and GenBank:EU205658] at 3 hours, four [GenBank:EU205815, GenBank:EU205658, GenBank:EU205357, and GenBank:EU205154] at 6 hours, six [GenBank:EU206217, GenBank:EU206830, GenBank:EU207063, GenBank:EU205154, GenBank:EU205815, and GenBank:EU205658] at 12 hours, one [GenBank:EU210321] at 24 hours, and one [GenBank:EU205580] at 48 hours post infection. Decreases in transcript abundance were also detected for three serpin transcripts [GenBank:EU204983, GenBank:EU205364, and GenBank:EU207110] at 3 hours, one [GenBank:EU207684] at 6 hours, three [GenBank:EU206929, GenBank:EU206496, and GenBank:EU207684] at 12 hours, and one [GenBanK:EU212079] in hemocytes at 24 hours post infection.

The serine protease transcript [GenBank:EU212480] with a detectable increase in abundance at 1 hour post infection shows high sequence similarity to the gene *Snake*, which is involved in the Toll Signaling pathway in *Drosophila *(*Drosophila *Flybase database, Revision 1.65, Sept. 10, 2005) [23]. At 6 and 12 hours post infection, one of the serine protease transcripts [GenBank:EU205154] that showed a detectable decrease in abundance had high sequence similarity to a *Bombyx mori *prophenoloxidase activating enzyme. Also at 6 hours post infection, there was also a detectable decrease in the abundance of a serine protease transcript [GenBank:EU205357] that has high similarity to the gene *Easter*, which is also involved in the Toll Signaling pathway in *Drosophila *(*Drosophila *Flybase database, Revision 1.65, Sept. 10, 2005) [23]. Finally, at 3 and 48 and 6 and 12 hours post infection, there was a detectable decrease in the relative abundance of two serine protease transcripts [GenBank:EU205580 and GenBank:EU205815, respectively] with known roles in the melanization response (*Drosophila *Flybase database, Revision 1.65, Sept. 10, 2005) [23].

### Anti-Microbial Peptides

At 3 hours post infection, there was a detectable increase in the abundance of a cecropin transcript [GenBank:EU205560] that shows sequence similarity to cecropins found in *Anopheles gambiae *[24]. At 6 hours post infection, there was a detectable increase in the abundance of two cecropin transcripts [GenBank:EU208659 and GenBank:EU210693] that show sequence similarity to cecropins in *Aedes aegypti *[24]. At 12 hours post infection there were two defensin transcripts [GenBank:EU205277 and GenBank:EU205824] showing a detectable decrease in abundance in response to filarial worm infection. There was also a detectable decrease in the abundance of two lysozyme transcripts [GenBank:EU206072 and GenBank:EU205505] that both have sequence similarity to lysozymes found in *An. gambiae *[24]. At 24 hours post infection, in the hemolymph, there was a detectable decrease in the abundance of a gambicin transcript [GenBank:EU206422] with sequence similarity to a gambicin found in *An. gambiae *[25]. It has been shown that cecropins are active against filarial worms *in vitro *[26], but this is the first report of cecropins, as well as other AMPs, showing transcriptional activity during an innate immune response to filarial worms *in vivo*, utilizing a natural system (i.e. no injection or wounding).

### Cytotoxic Reactions

There were multiple transcripts involved in the metabolism of reactive intermediates that showed significantly different transcriptional behavior as a result of filarial worm infection, thus facilitating the return to homeostasis after a successful immune response. Increases in transcript abundance were detected for an oxidoreductase transcript [GenBank:EU205969] at 3 hours, a glutathione transferase transcript [GenBank:EU206680] and two oxidoreductase transcripts [GenBank:EU212487 and GenBank:EU207343] at 6 hours, a cytochrome P450-9b2 transcript [GenBank:EU212874] at 24 hours, and a peroxidase transcript [GenBank:EU208780] and oxidoreductase transcript [GenBank:EU211957] at 72 hours post infection. Decreases in transcript abundance were detected for a glutathione S transferase transcript [GenBank:EU209900] at 1 hour; a superoxide dismutase copper chaperone transcript [GenBank:EU207555], an oxidoreductase transcript [GenBank:EU205803], and a glutathione synthase transcript [GenBack:EU208580] at 3 hours; an oxidoreductase transcript [GenBank:EU211799] at 12 hours; and an oxidoreductase transcript [GenBank:EU207215] at 24 hours post infection.

### Unknowns

**Figure 3:**
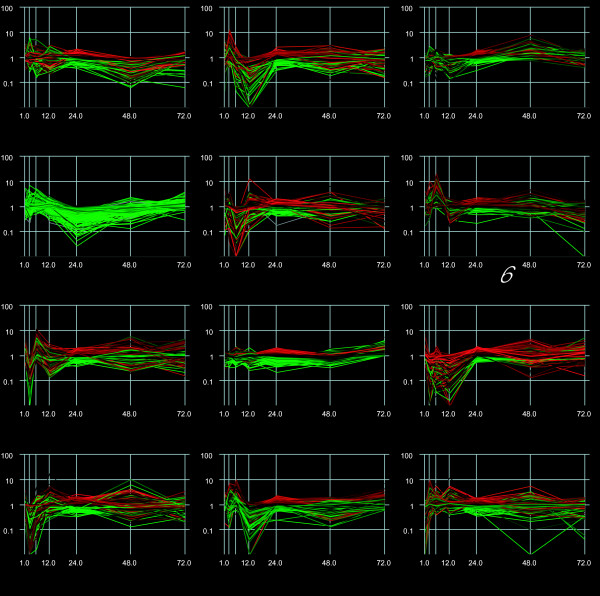
12 K-means Cluster Analysis of features changing in response to *B. malayi *infection (y-axis, log-scale) over time (x-axis) colored by significance (red = more significant; green = less significant). K-means clustering was used to group genes with similar transcriptional patterns. Cluster six (numbered) contains 517 features, including 26 features with known immune activity, e.g., AMPs, pattern recognition molecules, serine proteases, etc. Clusters were constructed so that the average behavior in each group was distinct from any other group. This serves as a means to implicate unknowns in processes based on temporal transcriptional behavior, and based on semblance to other transcripts/pathways that maintain a similar profile.

**Figure 4:**
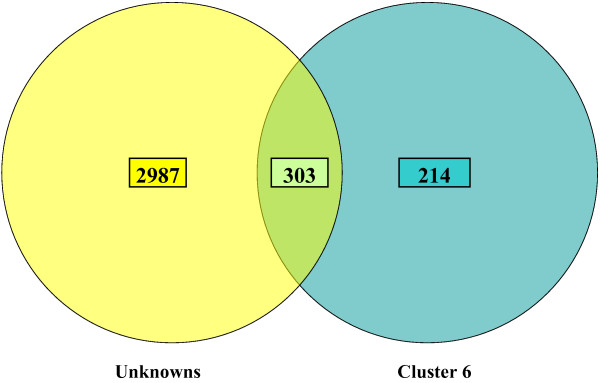
The number of transcripts with unknown function found within cluster six. Cluster six contained 517 features, 303 of which were unknowns or conserved unknowns. Of these 303 transcripts, 41 of them showed significantly different transcriptional behavior over the time course (at a 95% confidence interval over two-fold values). By narrowing down the number of candidates selected for further study, it becomes a very tractable problem to elucidate the probable function of a number of these unknown features. And, this can be accomplished by the mining of these unknown features based on similarities in transcriptional patterns with those of known genes.

K-means clustering was used to group genes with similar transcriptional patterns to begin to understand the temporal transcriptional behavior of unknown genes and find candidates for further analysis. An optimal cluster target of 12 was chosen (Fig 3). Clusters were constructed in which the average behavior in each group was distinct from any other group. Cluster six, containing 517 features, was interesting because it contained many immunity related transcripts. Of the immunity related transcripts, there were 9 AMPs (4 defensins, 4 cecropins, 1 diptercin), 6 serine proteases and 2 serpins, 5 cytotoxic molecules, 2 pattern recognition elements (C-type lectins), and 2 melanization specific molecules (DCE and a monophenol monooxygenase). This cluster also contained 303 transcripts with no previously described function (Fig 4). Of these 303 transcripts, 41 of them showed significantly different transcriptional behavior over the time course. By narrowing down the number of candidates selected for further study, it becomes a very tractable problem to elucidate the probable function of a number of these unknown features. And, this can be accomplished by the mining of these unknown features based on similarities in transcriptional patterns with those of known genes [27]. In addition, of the 761 transcripts that showed significantly different transcriptional behavior over the time course of the whole experiment, 427 of those transcripts were unknowns or conserved unknowns, suggesting that while the biosynthetic pathway of melanization is well understood [1], many factors involved in the anti-filarial worm immune response are not known.

### Hemocyte vs. Whole Body

**Figure 5:**
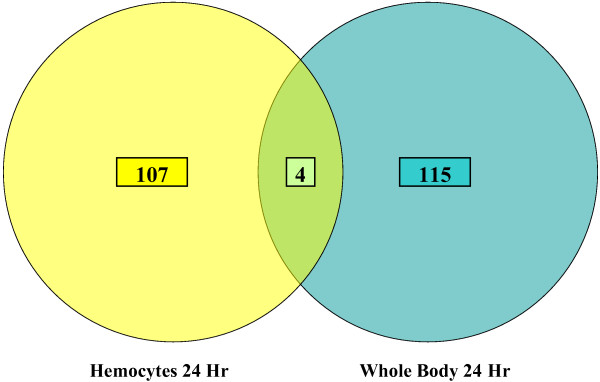
Shared transcripts between hemocyte vs. whole body RNA sources. Four transcripts with a significant detectable increase or decrease in abundance were shared between hemocyte RNA and whole Body RNA. 107 unique transcripts were attributed to hybridizations done with a hemocyte RNA source. 115 unique transcripts were attributed to hybridizations done with a whole body RNA source. Of the 4 shared transcripts, 1 is unknown [GenBank:EU206282], 2 are conserved unknowns [GenBank:EU205740 and GenBank:EU207864], and 1 is a protein kinase [GenBank:EU209290].

**Figure 6:**
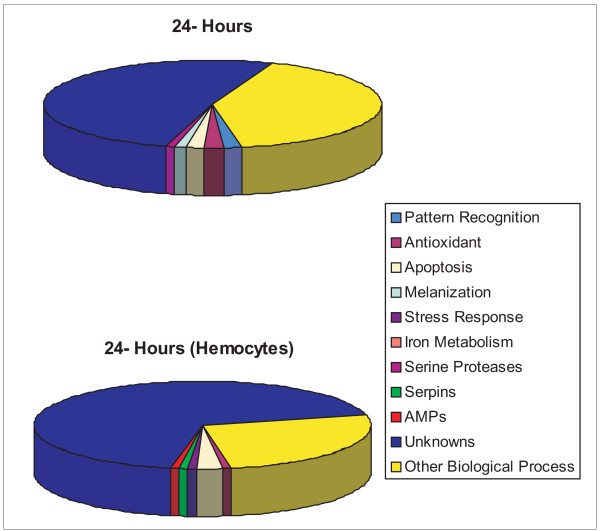
Functional composition of genes significantly affected by parasite challenge based on abundant and immunity-related EST clusters observed from *Ar. subalbatus *cDNA libraries. Transcripts showing significantly different behavior when array hybridizations were done with RNA generated from whole bodies (top) and hemocytes (bottom) 24 hours following parasite challenge.

Because the anti-filarial worm immune response primarily occurs in the hemocoel [28], hemocytes were collected at 24 hours post infective blood meal and compared to arrays hybridized with material generated from whole body collections at the same time. The two RNA sources, at this time point, yielded highly disparate results: 107 transcripts were attributed to hybridizations done with a hemocyte RNA source, and 115 transcripts were attributed to hybridizations done with a whole body RNA source. This reveals clues to the high tissue specificity of the anti-filarial worm immune response in *Ar. subalbatus *(Fig 5), and emphasizes the value of utilizing tissue-specific probes to conduct microarray studies. Of the transcripts that showed a detectable increase or decrease in abundance, only four were shared between the two RNA sources (an unknown [GenBank:EU206282], two conserved unknowns [GenBank:EU205740 and GenBanK:EU207864], and a protein kinase [GenBank:EU209290]) (Fig 6).

### Microarray Validation

Microarray data were confirmed using both *in silico *analyses of known transcriptional information in the literature and laboratory-based analyses via qPCR [29-31]. The transcriptional activity of a number of different response elements, induced by bacteria or filarial worms, has been characterized previously in several mosquito species. Based on this information, and the fact that the RNA used to screen the arrays in these studies result from the induction of a melanization-based immune response, it was expected that a number of parasite responsive elements on the arrays would show significant transcriptional patterns [7,19,32-35].

In conjunction with *in silico *validation of array results, qPCR provided independent, experimental verification of transcript abundance from the same total RNA used in the initial array experiment. Because the corroboration of all microarray data was impractical, a subset of nine genes was chosen for confirmatory studies. Transcriptional activity of the nine selected genes was verified at 1, 3, 6, and 12 hours post challenge, and five, four, three, and six of the nine genes, respectively, corroborated with transcriptional patterns detected on the array. Because of the method employed in selecting candidates for qPCR, it is not surprising that all genes do not corroborate at each time point. The elimination of a subset of genes from our dataset, at each time point, may reflect the differential sensitivities of the techniques or sample variation. This in turn, still, validated that the array was working as expected, showing all three conditions (increase, decrease, and no detectable change in transcript abundance). In addition, a number of "house-keeping" genes (ex. Ribosomal genes, actin, cytochrome C oxidase, etc.) included on the array showed no detectable change in transcript abundance throughout experimentation (data not shown), thereby providing further validation of the expression patterns detected. These results in combination with *in silico *and laboratory-based validation provided confidence that the transcriptional profiles are an accurate depiction of the biological phenomena under study.

### Verification of parasite infection

The average mean intensity of mf ingested in an infective blood meal was 56.1 ± 96.1 per mosquito. The average mean intensity of mf reaching the hemocoel was 15.5 ± 19.2 per mosquito. Of those mf that reached the hemocoel, 92.6% had some melanin deposited on them within 24 hours of infection. There was 100% prevalence of infection for all mosquitoes dissected.

## Discussion

To date, most studies examining the anti-filarial worm immune response in *Ar. subalbatus *have taken a reductionist approach, focusing on characterization of enzymes involved in melanization reactions (e.g., [19,34] or see [1] for review), and on characterization of proteins that have *in vitro *anti-microbial or anti-filarial worm activity [26,36]. Most of these previous studies have relied on intrathoracic injection of mf into the hemocoel to initiate the anti-filarial worm immune response, and numerous questions still remain regarding this response in *Ar. subalbatus*. To gain a more complete understanding of the intricacies of this response, as well as a more complete characterization of the players involved, a holistic approach that more closely mimicked the natural scenario needed to be engaged.

*Ar. subalbatus *is a medically important vector mosquito; however, there is little prospect for the sequencing of its genome in the near future. As a result, the EST libraries and microarrays used to conduct this study represent the only genomic tools available to holistically gauge immune responsiveness in this vector. This study represents the first attempt to holistically characterize the molecular components involved in the refractoriness of *Ar. subalbatus *to *B. malayi *following a natural blood feeding; therefore, it may give a more accurate depiction of the phenomena under study.

In the current study, the majority of genes previously implicated in the biosynthesis of melanin did not show significantly different transcriptional behavior, but this is not surprising considering the experimental design. This experiment was conducted under natural conditions, i.e. in the presence of a blood meal, and many of the intermediates involved in defense responses share the same biochemical pathways as intermediates involved in the physiologies associated with blood feeding (e.g., egg shell tanning and melanization) [37]. These molecules are probably regulated in response to immediate biological circumstances and are 'switched' on or off depending on the immediate need of the mosquito. Regardless of the physiological challenge, blood feeding induces dramatic changes in the transcriptome of the mosquito [38,39]. Consequently, there is the possibility of washing out the transcriptional activity of molecules induced by blood feeding that are used for both defense responses and physiologies associated with blood feeding.

For example, among the players involved in melanin biosynthesis in *Ar. subalbatus*, prophenoloxidase 2 transcript has been shown to increase in abundance in response to blood feeding [40]. Therefore, in the current experiment, it is possible that a significant increase in abundance of this transcript would not be evident due to the use of naïve blood fed mosquitoes as experimental controls. It also has been shown previously that knockout of proPO 1 inhibits melanization of mf in *Ar. subalbatus *[41]. However, that study was conducted using intrathoracic inoculation of *D. immitis *mf, in the absence of a blood meal, to stimulate the melanization response and an increase in the abundance of the transcript has only been shown following injection of *D. immitis *mf [40]. In addition, there is no transcriptional data available concerning AsproPO1 and *B. malayi*.

A similar study was conducted by Huang et al. (2005), showing that knockout of dopachrome conversion enzyme (DCE) dramatically reduced the melanization capacity of *Ar. subalbatus*. Again, that study was conducted using intrathoracic injection of *D. immitis *mf, in the absence of a blood meal, to initiate the melanization response. Furthermore, DCE transcript was not shown to have a significant increase in abundance in the mosquito whole body after injection of mf. In fact, it was not until 48 hours following mf inoculation that DCE transcript increased in abundance, and the transcript was most abundant in hemocytes [34]. Therefore, it comes as no surprise that there was no detectable increase in the abundance of DCE transcripts in whole-body material following challenge with *B. malayi*, lending credence to the possible high tissue specificity of this response and the value of using tissue specific probes when conducting microarray experiments.

In *Ar. subalbatus *it has been shown that RNAi knock-down of PAH significantly reduced melanization of inoculated *D. immitis *mf [19]. However, previous attempts to evaluate the transcriptional profile of PAH following feeding on *B. malayi *infected blood meals via northern analyses were inconclusive [19]. There has been no attempt to analyze the affect of PAH knock-down on the melanization of *B. malayi *mf *in vivo*. The rate-limiting substrate for melanin biosynthesis is tyrosine, and the sole source of endogenous tyrosine in mosquitoes is the hydroxylation of phenylalanine by PAH [19]. Although the blood meal may provide a sufficient amount of exogenous tyrosine for mosquito reproduction and other physiological processes, our transcriptional data suggest that PAH may provide an endogenous source of tyrosine to be used to combat filarial worm infection.

The biosynthesis of melanin in the open circulatory system of a mosquito is potentially very dangerous if the toxic by-products are not targeted to the surface of the pathogen. Previous studies have shown that melanin is specifically targeted to the surface of invading filarial worms, but the mechanisms and molecules involved in this targeting remain largely unknown [1]. Innate immune responses in insects are initiated by pattern recognition receptors (PRRs) that recognize microbial cell wall components such as peptidoglycan and β 1,3-glucan (GRP). Pattern recognition receptors then activate signaling cascades that regulate immune responsiveness [32]. A hemocyte-produced GRP from *Ar. subalbatus *has recently been characterized [32], which preferentially localizes to the area around the excretory pore of *B. malayi *mf [1], likely playing a role in the initiation of the melanization immune response. Lectins function as PRRs for both bacteria and filarial worms during the innate immune response of *Ar. subalbatus *as well as in other mosquito species [42,43]. Therefore, the peptidoglycan recognition protein, C-type lectins, and calreticulin detected in the current study may play roles in the anti-filarial worm immune response in *Ar. subalbatus*.

Further implicating calreticulin in this response are innate immunity studies done with other organisms. For example, calreticulin is present on the surface of hemocytes and is possibly involved in immunity-related phagocytosis in the cabbage moth, *Pieris rapae*, because blocking of calreticulin causes a decrease in the ability of hemocytes to phagocytize yeast cells [44]. Calreticulin also has been identified as a putative early-stage encapsulation response protein in *P. rapae*, playing a probable role in the non-self-recognition process. This is supported by the fact that blockage of calreticulin in this insect by a venom, calreticulin-like protein of endoparasitoid wasps, inhibited hemocyte spreading, attachment to the surface of the wasp, and subsequent encapsulation. Therefore, this parasite-specific protein might function as an antagonist, competing for binding sites with host calreticulin, to potentially suppress host immune responsiveness [45].

During melanin biosynthesis, phenoloxidase (PO) is normally present as inactive proPO. The inactive zymogen is cleaved into active PO by a serine protease, which is activated through the sequential cleavage and activation of other serine proteases [46]. Serine proteases and serpins modulate a diverse array of functions in insect innate immunity. They are involved in hemolymph coagulation, activation of AMP production, and melanin biosynthesis [47]; therefore, the large number of serine proteases and serpins showing significantly different transcriptional behavior in our study was expected. Serpins also could be involved in melanin localization, alone or in concert with pattern recognition receptors. When cleaved by a proPO activating enzyme, the cleaved serpin becomes inactive, initiating melanization. If the serpin active site contains a transition metal ion (Cu or Fe) that is exposed when inactive, the interactions of these ions with certain intermediates of oxygen and/or nitrogen will produce cytotoxic molecules (ROI and RNI, respectively) at the site of engagement. Subsequently, melanin would function, at least in part, to sequester the cytotoxic molecules. In this regard, melanization processes would be cytoprotective as well as cytotoxic [46,48].

In addition, melanin intermediates have cytotoxic functions, due in part to their ability to bind covalently to cell-membrane components and other cellular nucleophiles. This ability promotes redox-cycling, a process that promotes free-radical cascades and generates reactive intermediates of oxygen and nitrogen (ROI and RNI), producing a potentially harmful environment within the open circulatory system of an insect [48]. It has also been shown that blood feeding produces a chronic state of oxidative stress in a *Plasmodium-*resistant, melanizing strain of *An. gambiae *(L35), and the resultant increase in levels of reactive oxygen species increased the melanization of parasites [49]. Accordingly, mosquitoes must have a mechanism that protects them from the damaging effects of the oxidative stressors produced during melanization reactions. However, mosquitoes lack glutathione reductase, which many organisms rely on to reduce glutathione disulfide to harmless glutathione [1]. There have been many alternative enzymes identified (superoxide dismutases, peroxidases, catalases, thioredoxin reductases, and thioredoxin peroxidases) in the transcriptomes of *Anopheles *and *Aedes *that help mosquitoes tolerate and/or eliminate potentially harmful cytotoxic intermediates [7,50,51], but there are no data that specifically address the possible role these enzymes play during melanization immune responses.

There is evidence that *Ar. subalbatus *employs anti-oxidant molecules for both reactive oxygen species (ROS) production and for recuperative metabolism following an oxidative burst, and it has been hypothesized that ROI and RNI generated during melanin biosynthesis are the causative agents of death to sequestered pathogens [48]. Whatever the case, *Ar. subalbatus *must utilize a variety of mechanisms to reduce potentially harmful reactive intermediates, produced as a result of melanin biosynthesis, and to eliminate reactive intermediates hypothesized to be involved in the killing of sequestered pathogens. The current study provides information on a multitude of transcripts, potentially involved in the metabolism of reactive intermediates that showed significant differences in transcript abundance as a result of filarial worm infection, thereby revealing clues to the potential roles these enzymes play during melanization immune responses.

Anti-microbial peptides are effector molecules that are produced in the fat body, hemocytes, and epithelial tissues. They are considered a primary defense element in mosquito innate immunity, and increases in transcript abundance have been correlated not only with responses to bacteria and fungi [52,53], but also with stages of *Plasmodium *infection in *Anopheles *[25]. This is the first report of AMPs having transcriptional activity in response to filarial worm infection, *in vivo*, in *Ar. subalbatus*. Despite these correlations, our knowledge of the molecular mechanisms and the true role of these peptides in mosquito innate immunity remain limited.

There is little information in the literature (not involving cuticular damage, injection, etc.) on the effects of AMPs on the development of eukaryotic parasites *in vivo*. Defensin peptide has been detected in the hemolymph of mosquitoes inoculated with mf of *B. malayi *or *D. immitis *[54], and it has also been shown in previous studies that there is no defensin transcriptional activity in *Ar. subalbatus *fat body in response to *B. malayi *ingested 8 hours following a blood meal [36]. However, based on our array results, it appears that 8 hours post infective blood meal is too early to detect transcriptional activity of defensin in *B. malayi *challenged mosquitoes.

It has been suggested that the anti-microbial activity of AMPs might be an ancillary property, and that AMPs have other more important roles to play in innate immune activity [54]. This claim is substantiated by the fact that multiple hemolymph proteins seem to occur in complexes, suggesting that they exist in primed states as pre-immune complexes (e.g., AsGRP with PO or Defensin with PO), thereby limiting or not requiring immunity related transcriptional activity upon pathogen exposure [1,32,55]. However, their specific roles in targeting and/or activating the anti-filarial worm response remain unknown. The fact that transcriptional activity is minimal at earlier time points and that a decrease in abundance of the transcripts is occurring at later time points, suggests that defensin, as well as other AMPs, are functioning in a capacity beyond their proposed bactericidal role during the immune response.

The refractoriness elicited by *Ar. subalbatus *becomes increasingly interesting as one considers the recent studies that clearly verify the need to work with natural mosquito-parasite systems [33,56-58]. Studies of mosquito model systems have contributed significantly to our understanding of innate immunity and its role in vector competence; however, investigations of natural mosquito-parasite systems are critical because host-parasite interactions represent coevolved adaptations of significant complexity, and these relationships depend on the relative capacities of the host to recognize and respond to foreign invaders and of the parasite to weaken or suppress this response [7]. Without doubt, it is the genetic makeup of the mosquito that significantly contributes to vector competence; therefore, studies with mosquito model systems are conjectural at best. Innate immunity involving melanization does serve as a natural effecter of mosquito immune responses that mediate resistance or limit infection intensities of specific mosquitoes for specific parasites, eg., *Ar. subalbatus *and *B. malayi*, *Aedes trivitattus *and *D. immitis*, *Anopheles quadrimaculatus *and *B. pahangi *[16,21,28,59]. For that reason, comparing *Ar. subalbatus-B. pahangi *susceptibility and *Ar. subalbatus-B. malayi *refractoriness could provide significant insight into recognition mechanisms required to mount an effective anti-filarial worm immune response in the mosquito, as well as provide considerable detail into the molecular components involved in vector competence.

## Conclusion

This initial study provides a foundation and direction for future studies, which will more fully dissect the nature of the anti-filarial worm immune response in this mosquito-parasite system. It also serves as the underpinning to ask more complex questions and design more intricate experiments, including RNAi studies involving unknowns with significantly different transcriptional patterns. This in turn will help clarify the molecular components involved in the *Armigeres subalbatus-Brugia malayi *anti-filarial worm immune response. The study also argues for continued studies with RNA generated from hemocytes (and perhaps other tissues) and whole bodies to fully expound the nature of the anti-filarial worm immune response.

## Methods

### Mosquito Maintenance

*Ar. subalbatus *used in this study were obtained from the University of Notre Dame in 1986, and were reared and maintained as described previously [28].

### Exposure to infective bloodmeal

Sucrose pads were removed from mosquito cartons 14 to 16 hours prior to exposing mosquitoes to an infective bloodmeal. Mosquitoes were exposed to *B. malayi *(originally obtained from the University of Georgia NIH/NIAD Filariasis Research Reagent Repository Center (FR3)) by feeding on ketamine/xylazine anesthetized jirds, *Meriones unguiculatus *(microfilaremias of 40–180/20 μl). All animals and animal facilities are under the control of the School of Veterinary Medicine with oversight from the University of Wisconsin Research Animal Resource Center. Trained animal caretakers and veterinarians ensure proper care and handling of all animals based on internationally recognized guidelines. Forty mosquitoes were dissected at 0 and 24 hours post feeding to estimate the mean intensity of mf taken up in a blood meal, and the mean intensity of mf reaching the hemocoel. Control mosquitoes were exposed to naïve blood meals by feeding on anesthetized, uninfected jirds. Microfilaremias were determined from blood collected by orbital puncture. Mosquitoes that fed to repletion were separated into cartons and maintained in an environmental chamber.

### Material collection and RNA extraction

RNA was collected for microarray analysis from 3- to 4-day-old female *Ar. subalbatus *whole bodies with the heads, legs, and midguts removed (carcasses). Carcasses were collected at 1, 3, 6, 12, 24, 48, and 72 hours after blood feeding and stored at -80°C prior to RNA extraction. At each time point, *B. malayi *fed carcass RNA was compared to naïve blood fed carcass RNA. RNA was extracted from collected carcasses (groups of 10) via the single-step acid guanidinium thiocynate-phenol-chloroform extraction method [60]. RNA was purified using the Promega SV Total RNA Isolation System, which includes a DNase treatment step performed directly on the column membrane to reduce genomic DNA contamination. RNA was eluted from the column, dried using a speedvac, re-suspendend, and measured at 260 nm to determine RNA concentration of each sample.

In addition, RNA was obtained from hemocytes at 24 hours after parasite exposure and compared to RNA obtained from hemocytes 24 hours after a naïve bloodmeal. Hemocytes were perfused from 100 mosquitoes in both the experimental and control groups [61]. This time point was chosen because quality isolation of RNA from newly blood fed mosquitoes can be problematic, and collection of reasonable quantities of hemocytes by perfusion before the peritrophic matrix forms (~18 hours), is extraordinarily difficult. RNA was extracted from hemocytes via the single-step acid guanidinium thiocynate-phenol-chloroform extraction method and re-suspended in 20 μl of DEPC treated water. The samples were then DNase treated, re-extracted, and quantified in the same manner as carcass RNA.

### Microarray Design

Please note that the terminology used to define the components of the array are derived from the original DNA microarray paper [62]; therefore, the target is that which is tethered to the array substrate and the probe is the labeled material in solution that hybridizes to the target. Six cDNA libraries from adult female mosquitoes were used to generate 8,020 Expressed Sequence Tag (EST) clusters [53] from which 60-mer oligonucleotides were synthesized that represent 6,143 unique EST clusters. Oligos were designed using OligoArray v 2.1 [63], and these 6,143 oligonucleotides were used to construct our *Armigeres *microarray.

*Ar. subalbatus *microarray slides (Corning Ultragaps) were printed using BioRobotics 10 k pins with a BioRobotics Microgrid II arrayer (23°C and 50% humidity). Sixty-mer oligonucleotides were re-suspended at 40 μM in Pronto spotting buffer (Promega, Madison, WI). Each microarray (19,200 total tool spots) was printed using 16 pins, resulting in 48 subgrids (20 × 20 at 0.215 mm pitch). Features were printed in triplicate across the slide.

Cytochrome C oxidase was printed at all four corners of each of the 48 subgrids and served as a positive control. Negative controls on the array included blanks (spotting buffer with no oligo) and two mouse oligos, Mus 1 and Mus 2. Blanks were interspersed within all subgrids, and Mus 1 and 2 were printed in triplicate like all other features. A scanning control, Mus 3, was added because of its ability to be mixed with both Cy3 and Cy5 reactions, resulting in a 1:1 ratio on the array. Mus 3 RNA (2.5 ng) was added as a spike during cDNA synthesis.

Forty-eight spots appeared as empty, i.e. a spot touched down on the slide but visited an empty well for its source visit. These allow for new features to be added to the array without changing the general design. Overall, the array had 6,143 unique features printed in triplicate (= 18,429 spots, including Mus 1 and 2), 192 positive control spots, 48 Mus 3 spots printed once per subgrid, and 495 blank spots.

### cDNA Synthesis and Purification of amino allyl-modified cDNA

Complementary DNA synthesis was done according to the Pronto!™ *Plus *Indirect Labeling System with modification (use of an anchored oligo(dT) primer) (TM261, Promega). Priming with anchored oligo(dT) directed the start of synthesis from the 5' end of the poly-A tail. Ten μg of total RNA were used as a template for the synthesis of amino allyl-modified cDNA

### Coupling and Purification of CyDye labeled cDNA

Purified cDNA from each synthesis reaction was coupled to Cy3 or Cy5 according to manufacturers' instructions. The CyDye probes were purified using the ChipShot™ Membrane Clean-Up system (TM261, Promega). Purified cDNA was measured at 260 nm (Cy5 @ 650 nm and Cy3 @ 550 nm) to calculate yield. Calculation of cDNA yield was done using the Corning/Promega online cDNA calculator [64]. Probes (8–10 pmol/dye/slide) were dried down using a speedvac, resuspended at room temperature in 45 μl Pronto!™ hybridization buffer, incubated at 95°C for 5 minutes, and applied to the arrays. Arrays were hybridized overnight at 42°C and washed following manufacturers' specifications.

### Microarray analysis

The microarray data were prepared according to "minimum information about a microarray experiment" (MIAME) recommendations, deposited in the Gene Expression Omnibus (GEO) database, and can be accessed via the web [65]. All transcript and EST data for this project are publicly accessible in ASAP (A Systematic Annotation Package for community analysis of genomes) [24] as the complete collapsed set (ARALL v1) or through NCBI's GenBank database (accession numbers EU204979–EU212998) [66] via the web. Microarrays were scanned with an Axon GenePix 4000B (Molecular Devices, Foster City, CA) scanner and software using a 10 μm pixel size. Laser power was set at 100%, and the photomultiplier tube (PMT) was adjusted automatically to maximize the effective dynamic range and to minimize pixel saturation. Spot, size, location, and quality were determined using GenePix Pro 6.0. Potential misidentifications of spot quality and location were corrected manually. Three biological replicates, each with two technical replicates, done as dye swapped pairs (Cy5 experimental vs. Cy3 control) were performed for each experimental set in an effort to eliminate bias in the dye coupling [29,67]. Each biological replicate consisted of mosquitoes from distinct generations to take into account stochastic variations. Signal intensities were normalized using GeneSpring GX 7.3.1 software. All slides were normalized using a global linear regression (Lowess) curve fit to the log-intensity vs. log-ratio plot, and 20% of the data were used to calculate the Lowess fit at each point [68]. This curve was used to adjust the control value for each measurement. If the control channel was lower than ten, then ten was used instead. All data were averaged for replicate spots upon a slide, and then further averaged across slides. Minimum and maximum values were recorded and t-test p-values generated for all replicate sets. Genes showing differential expression over controls were isolated using volcano plots [69] at a 95% confidence interval over 2-fold values. Tests were parametric, but all variances were considered equal.

Data were clustered using K-means. K-means clustering is a non-hierarchical, unsupervised, non-deterministic, and iterative approach to grouping genes with similar expression profiles into clusters. Genes are partitioned into a user-specified number of clusters, K, so as to minimize 'within cluster' variability and maximize 'between cluster' variability. Thus, K-means clustering produces clusters of genes with a high degree of similarity within each cluster and a low degree of similarity between clusters [27]. Genes with no data in at least half of the starting conditions were discarded. The number of iterations was 1,000, using a Pearson correlation for a similarity measure, and an additional 100 random clusters also were tested.

Array analysis also involved examining genes according to Gene Ontology (GO) designations. Gene ontology designations were attached to EST cluster data in ASAP [24] and GeneSpring by migrating this information from the annotations of highly similar molecules in the *Drosophila *Flybase database (Revision 1.65, Sept. 10, 2005) [23]. Gene Ontology designations describe gene products in terms of their associated biological processes, cellular components, and molecular functions in a species-independent manner.

### Selection of candidate, differentially-expressed genes for validation

Three groups of candidate genes were selected from the 6,143 transcripts included on the microarray. Group 1 included the three transcripts with the highest degree of confidence at three hours (a glycine-rich secreted salivary peptide [GenBank:EU207085], a conserved unknown [GenBank:EU207715], and a potassium amino acid symporter [GenBank:EU210583]). Group 2 was a set of three transcripts selected randomly from those transcripts showing at least a 95% confidence interval over two-fold values (an unknown [GenBank:EU209612], a structural constituent of ribosome [GenBank:EU204988], and a DNA binding gene [Genbank:EU205805]). Group 3 was a set of three transcripts selected randomly from those transcripts showing, at most, a 25% confidence interval and were not necessarily designated as significant, as determined by the parameters expressed previously (troponin [GenBank:EU204998] and two additional unknowns [GenBank:EU206822] and [GenBank:EU211555]) (Christina Kendziorski, personal communication).

### Quantitative PCR

Transcript levels of the nine selected genes were measured using SYBR dye technology and quantitative PCR to validate microarray results. Complementary DNA was synthesized from 5 μg of total RNA prepared from RNA samples isolated for microarray analysis. Reverse transcription was done using MMLV Reverse Transcriptase (Promega, Madison, WI), 5 μg of total RNA, and an anchored oligo(dT) primer (2 μg/μl) (IDT, Coralville, IA). All amplifications and fluorescence quantifications were performed using an ABI 7300 Sequence Detection System and associated Sequence Detector Software v. 1.7 (Applied Biosystems, CA).

Primers were designed in Primer3 Software v. 0.3.0 [70] using default parameters with minor modifications (product size range 90–130 bp; optimum Tm 60; minimum Tm 57; maximum Tm 63). Primer pairs were selected in which at least three of the last five bases on the 3' end were adenine or thymine. Primer sets were validated to demonstrate that efficiencies of target and reference were approximately equal. In addition, primer sets were visualized via gel electrophoresis to verify amplicon size, sequenced to verify correct product amplification, and dissociation curves were run to determine any presence of primer dimer (data not shown). Fold differences for relative transcript abundance were calculated by the comparative C*t *method, using cytosolic small ribosomal subunit S17 as an endogenous control due to the fact that there was no detectable change in the relative abundance of the transcript during parasite challenge. Complementary DNA generated from naïve blood fed mosquitoes from the array experiments served as the calibrator [71].

## Authors' contributions

MTA validated results, analyzed data and prepared the text and figures. MTA and JFF infected mosquitoes, collected material, and conducted microarray experiments. GFM analyzed microarray data. CCC and BMC conceived this study and supervised all aspects of data collection and analysis.

## Supplementary Material

Additional file 1 Transcripts with a significant detectable change in abundance. A tabular representation of all transcripts showing a detectable increase or decrease in abundance at all time points. The table lists the gene name, associated product, GO ontology designation, and ASAPID number. All time points are represented, including transcripts showing a detectable change in abundance from microarrays hybridized with RNA collected from hemocytes. Click here for file
